# Plasma concentrations of tris(1-chloro-2-propyl) phosphate and a metabolite b*is(2-chloroisopropyl) 1-carboxyethyl phosphate* in Sprague-Dawley rats and B6C3F1/N mice from a chronic study of tris(chloropropyl) phosphate via feed

**DOI:** 10.1016/j.toxrep.2022.03.025

**Published:** 2022-03-29

**Authors:** Bradley Collins, Desmond Slade, Kristin Aillon, Matthew Stout, Laura Betz, Suramya Waidyanatha, Kristen Ryan

**Affiliations:** a National Institute of Environmental Health Sciences, Division of the National Toxicology Program, RTP, NC 27709-2233, USA; b MRIGlobal, Kansas City, MO 64110-2299, USA; c Social and Scientific Systems, Inc., Durham, NC 27703, USA

**Keywords:** Tris(chloropropyl)phosphate, Tris(1-chloro-2-propyl)phosphate, TCIPP, Bis(2-chloroisopropyl) 1-carboxyethyl phosphate, Sprague-Dawley rat, B6C3F1/N mouse, Systemic exposure, TCPP, Tris(chloropropyl)phosphate, TCIPP, Tris(1-chloro-2-propyl)phosphate, BCPCP, Bis(2-chloroisopropyl) 1-carboxyethyl phosphate

## Abstract

Tris(chloropropyl) phosphate (TCPP) is an organophosphorus flame retardant and plasticizer used in manufacturing and multiple consumer products. Commercial TCPP is a ubiquitous environmental contaminant and TCPP or its metabolites have been detected in human plasma and urine. In response to the demonstrated widespread human exposure and lack of toxicity data, the Division of the National Toxicology Program is investigating the chronic toxicity of TCPP following perinatal exposure in HSD:Sprague Dawley®SD® (HSD) rats (up to 20,000 ppm) and adult exposure in B6C3F1/N mice (females, up to 10,000 ppm; males up to 5000 ppm) to TCPP via feed. Systemic exposure and bioaccumulation were assessed by measuring plasma concentrations of tris(1-chloro-2-propyl)phosphate (TCIPP), the most abundant TCPP isomer. TCIPP concentrations in TCPP-exposed rats and mice ranged from 3.43 to 1180 ng/mL and increased with exposure concentration at all time points. No sex differences were observed in rats, but male mice had higher TCIPP concentrations than females. TCIPP did not bioaccumulate in rats or mice over the course of the study. Low TCIPP concentrations were seen in some control rats and mice that were attributed to background TCPP present during sample collection, preparation and/or analysis. Bis(2-chloroisopropyl) 1-carboxyethyl phosphate (BCPCP), a TCPP metabolite, was quantified in plasma from control and selected exposed animals. Results showed increases in BCPCP concentration that were proportional to exposure concentration in rats and mice at concentrations much higher than TCIPP, indicating that BCPCP might be a more suitable biomarker of TCPP exposure.

## Highlights

• Tris(chloropropyl)phosphate (TCPP) has widespread environmental exposure.
• We measured plasma levels of TCPP following chronic feed exposure in rodents.
• The most abundant isomer of TCPP (TCIPP) increased with exposure with no accumulation.
• Bis(2-chloroisopropyl)1-carboxyethyl phosphate (BCPCP) levels were higher than TCIPP.
• Metabolite BCPCP may be a more suitable biomarker for TCPP exposure.

## Graphical Abstract

**Figure:**
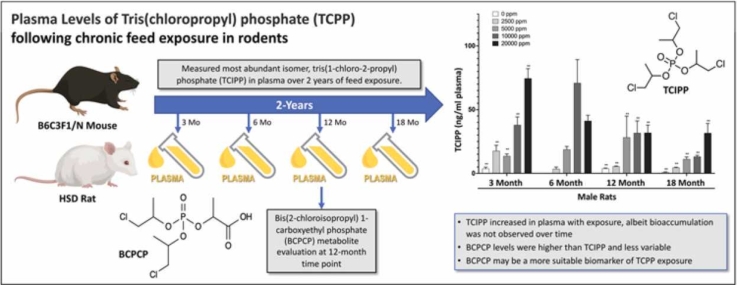

## 1 Introduction

**Fig. 1:**
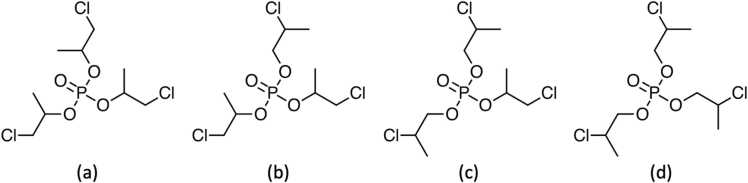
Structures of TCPP Isomers. (a) tris(1-chloro-2-propyl)phosphate (TCIPP, TCPP-1); (b) bis(2-chloro-1-methylethyl) 2-chloro-1-propyl phosphate; (c) bis(2-chloropropyl) 2-chloroisopropyl phosphate; (d) tris(2-chloropropyl) phosphate.

Tris(chloropropyl) phosphate (TCPP) is an organophosphorus flame retardant (OFR) utilized in numerous consumer products (i.e., mattresses, car seats, and upholstered furniture) and construction materials (i.e., rigid polyurethane foam, electronic products, coatings, etc) [2], [26]. Commercial TCPP is a mixture of four isomers identified in this document as: tris(1-chloro-2-propyl)phosphate (TCIPP); bis(2-chloro-1-methylethyl) 2-chloropropyl phosphate; bis(2-chloropropyl) 2-chloroisopropyl) phosphate; and tris(2-chloropropyl) phosphate. Of these, TCIPP is the most abundant isomer, representing 50–85% of the mixture (Fig. 1).

Due to its widespread use in consumer and commercial products, TCPP has been identified in many built environments, including laboratories and air handling systems [10], indoor dust [7], [20], [17], indoor air [15], and in surface waters [22] which suggests that the general population or workers may be exposed through a variety of routes including inhalation of vapors or particulates, direct skin contact, and incidental ingestion. In a recent report by National Institute for Occupational Safety and Health (NIOSH), TCPP was detected in samples associated with carpet installation, foam manufacturing and use, nail salons, roofing, and various other occupations [13], [12]. To better estimate human exposure in these scenarios, TCPP was also quantified in personal air samples and hand wipes of workers: Concentrations ranged from 30.2 to 87.1 ng/m^3^ in personal air samples and 27.3–106 µg/sample in hand wipe samples and were the highest of any flame retardant analyzed. In a review article, Hou [16] presents data summarizing inhalation exposures to OFRs which showed human TCPP exposures ranging from 3.0 to 10.0 ng/kg body weight/d in toddlers, 1.41–43 ng/kg body weight/d in children, and 0.5–4.1 ng/kg body weight/d in adults from 10 countries. These and many other reports highlight that TCPP is a widely used product, is ubiquitous in the environment, and that human exposures are occurring.

With the growing evidence for human exposure to TCPP, there is increased interest in assessing the risks to consumers’ health and safety related to the knowledge that structurally similar flame retardants, TDCPP (tris(1,3-dichloro-2-propyl) phosphate) and TCEP (tris(2-chloroethyl) phosphate), have been classified as carcinogenic [27], [21], [1], [19]. Toxicity data on TCPP has been summarized in reports by several regulatory agencies and organizations [1], [2]. Taken together, the experimental in vitro and in vivo data in these reports suggest that TCPP is not acutely toxic or genotoxic but repeat exposures to TCPP or its metabolites may adversely affect liver or kidney function or lead to developmental and/or reproductive toxicity. However, several data gaps still exist including an understanding of the carcinogenic potential of TCPP as well exposure kinetics [11], [14].

There are few absorption, distribution, metabolism, and excretion (ADME) or toxicokinetic (TK) studies in animals or human subjects reported for TCPP in the literature. A comparative ADME study of brominated and chlorinated phosphorylated flame retardants in rats, that included TDCPP and TCEP, showed that tris- or bis-dihalogenated compounds were more slowly absorbed, distributed and excreted than mono-halogenated compounds and that biliary excretion of chlorinated compounds was more rapid than that of brominated compounds [18]. Studies in human liver microsomes and hepatocytes show that TCPP is metabolized via phase 1 metabolism, including deesterification to produce isomers of 1-chloro-2-propyl phosphate (MCPP) and bis(1-chloro-2-propyl)phosphate (BCPP, Supplemental Fig. S1) and oxidation to produce three isomers of bis(1-chloro-2-propyl) 1-carboxyethyl phosphate (BCPCP, Supplemental Fig. S1), and isomers of bis(1-chloro-2-propyl)1-hydroxy-2-propyl phosphate (BCIPHIPP) [24], [23], [25]. BCPP and BCIPHIPP, biomarkers of TCPP exposure, have been identified in urine from children at concentrations up to 5.92 ng/mL and 12.8 ng/mL, respectively [4], and in intensive care patients at concentrations up to 10.3 and 37.2 ng/mL [5], which the authors attribute to exposure to indoor dust and plasticizers in medical equipment and tubing. BCPP and BCIPHIPP were also detected in urine of urethane foam applicators who were monitored for TCPP exposure: Mean concentrations of BCPP and BCIPHIPP were found to be 6.2 and 88.8 µg/mL, respectively, equivalent to ~30 times the average concentration in the general population [6]. Despite the available data, quantification of TCPP or its metabolites in biological matrices are rarely reported in a comprehensive manner, which complicates attempts to associate exposure with adverse health effects such as cancer.

To learn more, the Division of the National Toxicology Program (DNTP) is currently investigating the toxicity of TCPP in rodent models to provide data for this chemical [(Testing Status)]. Following perinatal TCPP exposure in HSD:Sprague Dawley®SD® (HSD) rats and adult B6C3F1/N mice, systemic exposure was assessed at multiple time points during a 2-year study using a validated analytical method to quantify TCPP in plasma from rats and mice using tris(1-chloro-2-propyl)phosphate (TCIPP or TCPP-1) as a marker for TCPP concentration [10]. During the development and validation of the analytical method used here, low levels of TCIPP were detected in control rat and mouse samples at some time points, which may reflect ubiquitous background levels of TCPP [10]. Because BCPCP arises from oxidation via Phase 1 metabolism, we explored the presence of BCPCP in the plasma of study animals to determine if it could potentially serve as a marker for in-life TCPP exposure. To that end, we qualified a liquid chromatography tandem mass spectrometry (LC-MS/MS) method to quantify the three isomers of BCPCP in HSD female rat plasma and used it to analyze selected study samples.

## 2 Materials and methods

### 2.1 Materials

TCPP (CASRN: 13674–84–5) was obtained from Albemarle Corporation (Baton Rouge, LA). The identity of the test material and its isomeric composition were previously published [10]. The purity of the lot used in the study (M072911NP) was determined to be approximately 97% based on the combined peak areas of the four identified TCPP isomers (Fig. 1). Over the course of the study the purity of the test material was confirmed 8 times and was found to be ≥ 97.7% relative to a frozen reference standard stored at − 20ºC.

The internal standard (IS), tripentyl phosphate (CASRN: 2528–38–3), used for plasma sample analysis for TCPP, was obtained from Tokyo Chemical Industry Co., Ltd., Tokyo, Japan).

BCPCP (CASRN: 1452871–14–5) was synthesized by MRIGlobal (Kansas City, MO) using a 3-step process involving reaction of ethyl lactate with phosphorus oxychloride (Supplemental Files, BCPCP Synthesis Method), which resulted in a mixture of three BCPCP isomers: bis(1-chloro-2-propyl) 1-carboxyethyl phosphate (BCPCP-1); (1-chloro-2-propyl) (2-chloro-1-propyl) 1-carboxyethyl phosphate (BCPCP-2); and (1-chlobis(2-chloro-1-propyl) 1-carboxyethyl phosphate (BCPCP-3) (Supplemental Fig. S1). The identity of BCPCP was confirmed by ^1^H-, ^13^C-, and ^31^P NMR and direct infusion mass spectrometry and the purity after derivatization with methyl iodide in acetonitrile, was based on a gas chromatography with flame ionization detection method (GC-FID, Supplemental Table S1, Fig. S2) reported by Chang [9], and found to be 95.7% as the sum of the 3 isomers with an isomer ratio of 55.0:29.3:15.7 for BCPCP-1, BCPCP-2, and BCPCP-3. The internal standard (IS) for BCPCP quantitation, dibenzyl phosphate (CASRN: 1623–08–1) was obtained from Sigma-Aldrich, St. Louis, MO.

Control male and female HSD rat and B6C3F1 mouse plasma used in calibration curves and quality control (QC) samples for both TCIPP and BCPCP analysis in plasma, was obtained from BioIVT (Westbury, NY). All other chemicals and reagents were procured from commercial sources.

### 2.2 Animals and husbandry

This study was conducted at Battelle (Columbus, OH). All research was approved by Institutional Animal Care and Use Committee and the study was conducted in compliance with the Food and Drug Administration Good Laboratory Practice. Animals were housed in facilities that are fully accredited by the Association for Assessment and Accreditation of Laboratory Animal Care International and procedures were in accordance with the “Guide for the Care and Use of Laboratory Animals” [34].

Time-mated female HSD rats were supplied from Harlan (Indianapolis, IN) to provide pups for the chronic exposure study. Dams were 11–15 weeks of age upon arrival and singly housed in solid bottom polycarbonate cages through gestation and weaning. All cages were bedded with irradiated Sani-chips® hardwood bedding (PJ Murphy Forest Products Corp, Montville, NJ). Animal room conditions were maintained at 72 °F ± 3 °F with humidity ranging from 50% ± 15%, and a 12-h light/dark cycle. Animals had ad libitum access to municipal tap water (Columbus, OH) and irradiated NIH-07 (Zeigler Brothers, Gardners, PA) diet during this phase. At weaning, offspring were group housed (up to 2 males or 4 females per cage) and were supplied ad libitum access to municipal tap water and NTP-2000 meal feed (Zeigler Brothers, Gardners, PA) for the remainder of the study.

B6C3F1/N mice were supplied by Taconic Farms (Germantown, NY) at 3–4 weeks of age. All cages were bedded with irradiated Sani-chips® hardwood bedding. Animal room conditions were maintained at 72 °F ± 3°F with humidity ranging from 50% ± 15%, and a 12-h light/dark cycle. Animals had ad libitum access to municipal tap water (Columbus, OH) and irradiated NTP-2000 meal feed (Zeigler Brothers, Gardners, PA). Male mice were housed individually, and females were grouped housed, up to 4 per cage.

### 2.3 Methods

#### 2.3.1 Study design and sample collection

The DNTP chronic toxicity study of TCPP in rats and mice comprised 2 cohorts of each species. Cohort 1 animals were used for the evaluation of general toxicity parameters and to assess histopathology for TCPP-related neoplasms. The cohort 1 study results are under review by the DNTP and data are publicly available: https://ntp.niehs.nih.gov/go/peerreview. Cohort 2 animals, exposed to the same TCPP concentrations and monitored at the same time as cohort 1, were used specifically for the evaluation of systemic exposure to TCPP. The data presented in this paper are from cohort 2 animals only. Additional details regarding these study animals and results are available at the NTP webpage: https://doi.org/10.22427/NTP-DATA-002-03220-0026-0000-7.

Pregnant rats were randomized into five treatment groups using a body weight-partitioning algorithm using PATH/TOX SYSTEM software (Xybion Medical Systems Corporation, Cedar Knolls, NJ) on gestation day (GD) 5. Feed formulations containing TCPP at concentrations of 0, 2500, 5000, 10,000 and 20,000 ppm, or blank feed, were provided throughout gestation and lactation. Feed formulations, including controls, were analyzed prior to administration and in animal room samples post-administration using a validated GC-FID method (Supplemental Table S2, Fig. S3) based on the summed peak areas of the TCPP isomers (r = 1.00; relative error (RE) ≤ ± 1.0%; relative standard deviation (RSD) ≤ 1.8%). All seven pre-administration and 3 of 5 post-administration measurements were within ± 10% of the target concentration. At birth, litters within each treatment group were standardized to a maximum of eight pups per litter (preferably 4 pups/sex) on postnatal day (PND) 4. On PND 28, 5 dams from each treatment group and their offspring (1 male and 1 female) were randomly selected for plasma collection and determination of TCIPP concentrations. Blood was collected from dams and pups into tubes containing K_3_EDTA as the anticoagulant and ~0.35 mL plasma was obtained via centrifugation. Following blood collection, dams and pups were humanely terminated by CO_2_ inhalation and properly disposed without further evaluation. The remaining offspring were weaned on PND 28, dams humanely euthanized, and 10 rats/treatment group/sex were assigned for plasma collection at 3, 6, 12, and 18 months of age (Note: the day after weaning was assigned Study Day (SD) 1). Feed containing TCPP was provided continuously during this time course. Blood samples were obtained from the retroorbital plexus and dispensed into K_3_EDTA tubes and plasma was isolated from blood by centrifugation. Following blood collection for the last time point (i.e., 18-month), rats were terminated via CO_2_ inhalation and disposed of properly without further evaluation. Plasma samples from all time points were immediately frozen and then stored at approximately –70 ºC until analysis.

Male and female rats assigned to the plasma collection group were monitored routinely for general toxicity measures. Mortality/moribundity was evaluated daily. Individual body weights were recorded for all weaned offspring on SD 1, once weekly for the first 3 months at 4-week intervals thereafter, and at removal from study. Clinical observations (formal, out-of-cage) and food consumption measurements were performed at intervals to coincide with body weight collections. Food consumption estimates were calculated over a 7-day consumption period and reported as the grams of feed consumed per animal. Based on food consumption, compound consumption (i.e. chemical intake) was estimated and reported as mg TCPP/kg body weight/day.

Male and female mice on study were provided feed formulations containing TCPP at concentrations of 0, 1250, 2500, 5000 ppm (males) or 0, 2500, 5000 and 10,000 ppm (females), or blank feed, throughout the time course. Formulations, including controls, were analyzed prior to administration and in animal room samples post-administration using the same GC-FID system as rats. All seven pre-administration formulations and 5 of 5 post-administration measurements were within ± 10% of the target concentration. Mice were stratified to four treatment groups using a body weight-partitioning algorithm. As in rats, mice were monitored for signs of toxicity including survival, clinical observations, body weights, and food consumption. Compound consumption was determined as in rats. Twenty mice/sex/treatment group were selected for plasma collection and TCPP quantitation. After 3, 6, 12, and 18 months on study, 5 males and 5 females from each treatment group were randomly selected for blood collection from the retro-orbital sinus into K_3_EDTA tubes. After blood collection, mice were terminated via CO_2_ inhalation and disposed of properly without further evaluation. Plasma was isolated from blood via centrifugation and maintained frozen on dry ice or in liquid nitrogen. Plasma samples were frozen at approximately –70ºC until analysis.

### 2.4 Sample preparation

Study plasma samples for rat time points < 6 months and mouse time points < 12 months were thawed, vortex mixed and 50-µL aliquots were transferred to individual vials. Each vial was spiked with 5 µL of ethanol, vortex-mixed and then treated with trichloroacetic acid (TCA, 10 µL). For rat time points ≥ 6 months and mouse time points ≥ 12 months, samples were prepared analogous to earlier time points with the exception that half of the aliquots were spiked with TCA prior to analysis for TCIPP, and the other half were spiked with acetonitrile (ACN, 100 µL), prior to BCPCP analysis, as described below. All aliquots were vortex-mixed and allowed to stand for ~15 min.

### 2.5 TCPP analysis

TCIPP concentrations were determined using a validated method employing gas chromatography coupled with flame photometric detection (GC-FPD) method [10]. TCIPP standards were prepared using TCPP, and the percent composition of TCIPP (67.57%) in TCPP determined during the purity assay.

Briefly, matrix standards at concentrations of 5 – 70 ng TCIPP/mL were prepared by spiking control plasma with 20 µL of an appropriate TCPP spiking solution prepared in ethanol from alternating stocks. Matrix quality control (QC) standards were prepared at 10 and 50 ng/mL by spiking 6.0 mL of pooled control plasma with 60 µL of an appropriate TCPP spiking solution prepared from alternating stock solutions. A 50-µL aliquot of each matrix standard was extracted by adding 250 µL of toluene to each vial, mixing, followed by centrifugation for 5 min at 2800× *g*. The supernatant was transferred to a 1-dram vial and the sample was extracted with a second 250-µL aliquot of toluene. The combined supernatant was evaporated to dryness using an N-EVAP (Organomation Associates, Inc, Berlin, MA) at 25 °C and the residue was reconstituted with 200 µL of toluene containing 40 ng/mL tripentyl phosphate (TPP) internal standard (IS). Study samples were prepared and analyzed similarly to matrix standards except that 20 µL of ethanol was used in place of TCPP spiking solutions. All samples were analyzed by GC-FPD.

All plasma sample and standard curve concentrations are reported as ng TCIPP/mL plasma. TCIPP concentrations were calculated from the matrix standard curve using a linear regression with 1/x weighting after correcting TCPP concentrations for TCIPP content of 67.57%, The analytical method was shown to be linear over the range of 5–70 ng TCIPP/mL (r ≥ 0.99). Matrix blanks and QC standards were analyzed throughout the analytical batch as a check on system performance. Sample runs were reevaluated when the %RE of QC standards exceeded ± 20%. Regression equations for matrix standard curves run with the samples were compared to 95% confidence intervals of a matrix curve control chart created from matrix standard curves prepared during method development and validation. When the slope and/or intercept for a standard curve fell outside the 95% confidence interval the standard curve was rejected and all samples were rerun.

Representative matrix and solvent calibration curves are shown in Supplemental Fig. S4. Typical chromatograms of a matrix standard, study sample, and a matrix blank are shown in Supplemental Fig. S5. The limit of quantitation (LOQ) and limit of detection (LOD), defined as 3 times the standard deviation of the low standard, were ~5 and ~1 ng/mL, respectively [10].

### 2.6 BCPCP analysis

Plasma samples were extracted using a solution prepared by diluting the internal standard (IS), dibenzyl phosphate, in methanol to a final concentration of 500 ng/mL. Matrix standards were prepared over the range of 30–10,000 ng/mL by spiking 100-µL aliquots of control female HSD plasma with 10 µL of an appropriate BCPCP spiking solution prepared in methanol and mixing for ~1 min. Matrix QC standards were prepared at concentrations of 100 and 5000 ng/mL by spiking 1.0 mL aliquots of control plasma with an appropriate BCPCP spiking solution prepared in methanol and mixing for ~1 min followed by addition of 200 µL of acetonitrile and 90 µL of extraction solution containing IS. Each sample was mixed for 30 s, centrifuged at 16,000 × *g* for ~10 min and the supernatant was filtered through a Phree™ phospholipid removal tube (Phenomenex Inc., Torrance, CA). The filtrate of each matrix standard was split into two aliquots and analyzed at the beginning and end of each batch. Matrix blanks and QC samples were analyzed throughout the analytical batch to assess system performance.

Aliquots pretreated with acetonitrile from female rat and male mouse controls at 6-, 12- and 18-month (rat) or 12- and 18-month (mouse) time points and from all exposure groups at the 12-month time point were evaluated for BCPCP to provide an additional assessment of system exposure. Each aliquot was extracted by adding 45 µL of extracting solution, vortex mixing followed by centrifugation at 16,000 × *g* for 10 min. Supernatant from each sample was filtered through a Phree™ phospholipid removal tube, and the filtrate was analyzed using by the LC-MS/MS system described in Supplemental Table S3. Instrument response was determined by combining the peak area of all three BCPCP isomers. BCPCP concentrations were calculated from the response of matrix standards using a linear regression with 1/x^2^ weighting.

## 3 Results

All study data are available in the Chemical Effects of Biological Systems (CEBS) database and can be accessed using the following link (https://doi.org/10.22427/NTP-DATA-002-03220-0026-0000-7). Specific tables are: Rat and Mouse E03, Growth curves; E04, Mean body weight summary; E05, Clinical observation summary; E08, Mean feed and compound consumption; PA48, Summary of tissue concentration; and IAD48, Plasma and TCIPP concentration for individual animals and are referenced in square brackets throughout this section.

### 3.1 Body weights and chemical consumption

#### 3.1.1 Rats

Survival of male and female rats was not affected by exposure to TCPP. Additionally, clinical signs indicative of exposure-related toxicity were not observed in any exposure group ([Rat E05]). At the earliest time point of plasma collection, PND28 (i.e., weaning), body weights of dams were unaffected by TCPP exposure (data not shown). However, male and female offspring at this time point exhibited lower body weights in a TCPP exposure-dependent manner. These changes were attributed to lower body weight gains rather than body weight loss during the lactation period. As such, body weights were 15% and 26% lower than controls for male offspring, and 12% and 27% lower for female offspring in the respective 10,000 and 20,000 ppm groups by start of the chronic study (SD 1) ([Rat E04]). This trend in body weights continued throughout the course of the chronic study and mean body weights for males in the 10,000 and 20,000 ppm groups were 7% and 9% lower than controls, respectively, at study termination ([Rat E03, E04]). Similarly, female body weights were lower than controls throughout the course of the study and were 13%, and 20% lower than controls in the 10,000 and 20,000 ppm groups, respectively, at study termination ([Rat E04]).

Food consumption ([Rat E08]) was evaluated in TCPP-exposed rats over the course of the chronic study. Consumption ranges randomly increased or decreased over controls with no clear pattern over time e.g., ranging from 40% lower than controls to 23% higher than controls at various time points across exposures groups), which is commonly observed in feed studies. Average feed consumption in males at the end of the study were 97%, 109%, 111%, and 91% of control values in the 2500, 5000, 10,000, and 20,000 ppm TCPP groups, respectively. Average feed consumption in females at the end of the study were 100%, 102%, 135%, and 75% of control values in the 2500, 5000, 10,000, and 20,000 ppm TCPP groups, respectively. In general, chemical consumption ([Rat E08]) was estimated to be approximately linear with the increasing exposure concentration in both males and females over the course of the study. Estimated TCPP consumption at the end of the study was 124.2, 276.6, 604.8, and 1017 mg/kg/day in males in the 2500, 5000, 10,000, and 20,000 ppm groups, respectively. Estimated TCPP consumption in females was 145.0, 305.2, 870.5, and 1057 mg/kg/day in the 2500, 5000, 10,000, and 20,000 ppm groups, respectively.

#### 3.1.2 Mice

Survival was not adversely affected by exposure to TCPP; all mice survived until their scheduled timepoints apart from one male in the 0-ppm group and 2 males in the 1250 ppm group which were recorded to have natural deaths. In addition, male and female TCPP-exposed mice did not display clinical signs of toxicity ([Mouse E05]). Body weights for male mice in the 2500 ppm and 5000 ppm groups were lower than controls over the course of this study. At study termination, mean body weights in each of these groups were 9% and 15% lower than controls, respectively ([Mouse E04]). Female body weights were also lower than controls throughout the course of the study and final mean body weights were 6%, 14%, and 36% lower in the 2500, 5000 and 10,000 ppm groups at study termination, respectively ([Mouse E03, E04]). Lower weights for both males and females are interpreted to be the result of lower body weight gains rather than body weight loss during the exposure period.

As seen in rats, food consumption ([Mouse E08]) for TCPP-exposed males and females was variable, showing random increases or decreases over controls within the 2-year exposure period. Average feed consumption in males at the end of the study was 115%, 111%, and 96% of control values in the 1250, 2500, and 5000 ppm TCPP groups. Average feed consumption in females at the end of the study was 84%, 108%, and 110% of control values in the 2500, 5000, and 10,000 ppm TCPP groups. Overall, the consumption was estimated to be approximately linear with the increasing exposure concentration in both males and females over the course of the study. TCPP consumption ([Mouse E08]) at the end of the study was 124.2, 276.6, and 638.8 mg/kg/day in males of the 1250, 2500, and 5000 ppm groups, respectively. In females, estimated TCPP consumption at the end of the study was 230.5, 649.0, and 1764.0 mg/kg/day in the 2500, 5000, and 10,000 ppm groups, respectively.

### 3.2 Plasma TCIPP concentrations

#### 3.2.1 Rats

**Fig. 2:**
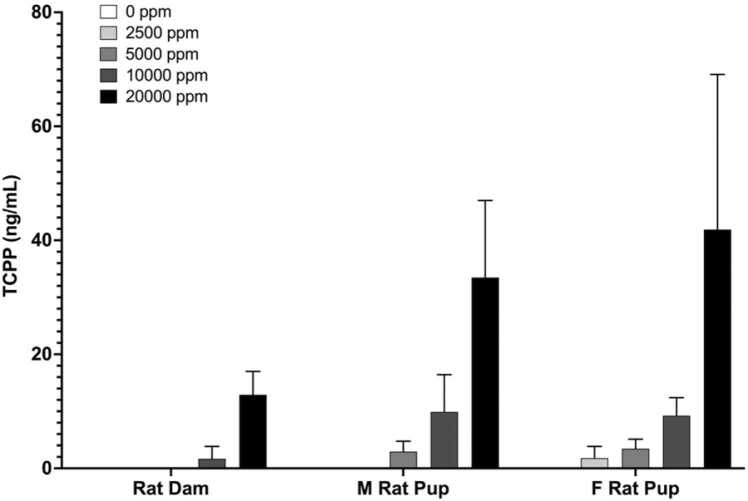
Mean TCIPP plasma concentrations in rat dams, and male and female rat pups at PND28. M=male; F=Female. Data shown: mean ± sd; n = 4–5. Note: Statistics were performed but because all controls were BLOD no significance could be assigned.

Plasma collected from rat dams and male and female offspring on PND28 were analyzed for TCIPP concentrations using the procedure described above. In general, TCIPP concentrations increased with exposure concentration in PND28 dams and offspring. No trend or pairwise statistical tests were performed on this data because the TCIPP concentrations in control dams were below the limit of detection (LOD, 0.94 ng/mL). Mean concentrations of TCIPP in PND28 dam plasma were also below the LOD in groups exposed to 2500 and 5000 ppm but were 1.68 and 12.9 ng/mL in the 10,000 and 20,000 ppm groups, respectively ([Rat PA48]; Fig. 2). Mean concentrations of TCIPP in male rat pup plasma ranged from below the LOD in the 2500 ppm exposure group to 33.5 ng/mL at the highest exposure concentration with an apparent increasing trend with exposure concentration. In female pup plasma, mean TCIPP concentrations ranged from 1.78 to 41.9 ng/mL at all exposure concentrations with an apparent increasing trend. TCIPP concentrations in male and female pup plasma were similar and tended to be higher compared to their dams.

**Fig. 3:**
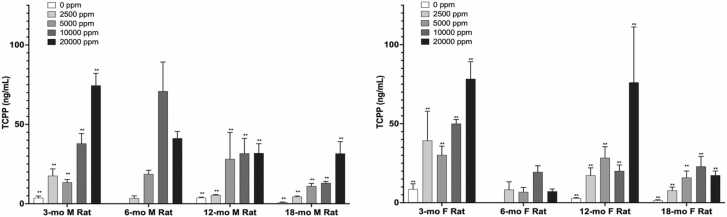
Mean TCIPP plasma concentrations in adult male and female rats at 4 time points during the 2 year study. M=Male; F=Female. Statistical analyses performed by Jonckheere (trend) and Shirley or Dunn (pairwise) tests. Statistical significance for the control group indicates a significant trend test. Statistical significance for a treatment group indicates a significant pairwise test compared to the vehicle control group. *Statistically significant at p ≤ 0.05; * *Statistically significant at p ≤ 0.01. Data shown: mean ± sd; n = 7–10.

Plasma was collected from adult male and female F1 rats at four time points (3, 6, 12, and 18 months) and was analyzed upon receipt using the procedure described above. Mean TCIPP exposure concentrations for each group at each time point are shown in ([Rat PA48]). Mean TCIPP concentrations in TCPP-exposed rats ranged from 3.43 to 74.5 ng/mL in males and 7.21 – 78.4 ng/mL in females and generally increased with exposure concentration at each time point, except at 6 months in both male and female rats, 12-months in male rats, and 18-months in female rats (Fig. 3). Concentrations of TCIPP in exposed animals were higher than controls in all exposed rat groups. TCIPP concentrations in TCPP-exposed male and female rats 3-, 12-, and 18-month time points) were significantly different from controls (p ≤ 0.01) and demonstrated a statistically significant increasing trend with TCPP-exposure (p ≤ 0.01). An increasing trend was also observed at the 6-month time point, but it was not statistically significant. Within a timepoint, there appeared to be no difference in TCIPP concentrations between males and females. In male rats, over the course of the study, mean TCIPP concentrations ranged from 17.6 to 4.46 ng/mL, 13.6 – 11.2 ng/mL, 38.0 – 13.1 ng/mL, and 74.5 – 31.6 ng/mL in the 2500, 5000, 10,000 and 20,000 ppm exposure groups, respectively. TCIPP concentrations for each exposure group were similar to each other at 3 and 12 months and 6 and 18 months but varied between time points, with the 6- and 18-month time points exhibiting consistently lower TCIPP concentrations at all exposure groups, except the 10,000-ppm group at 6-months, than the same exposure groups at 3- and 12-months. In male rats, no consistent increasing trend in TCIPP concentration was observed with study duration, indicating a lack of bioaccumulation over the course of the study. Mean concentrations of TCIPP in female rats ranged from 39.3 to 7.75 ng/mL, 30.2–15.9 ng/mL, 50.0 –23.0 ng/mL and 78.4–17.4 ng/mL in the 2500-, 5000-, 10,000- and 20,000-ppm exposure groups, respectively ([Rat PA48]). The pattern of TCIPP concentrations in females between time points over the course of the study was similar to males, with concentrations at 6 and 18 months tending to be lower than those at 3 and 12 months with no consistent increases observed over the course of the study.

#### 3.2.2 Mice

**Fig. 4:**
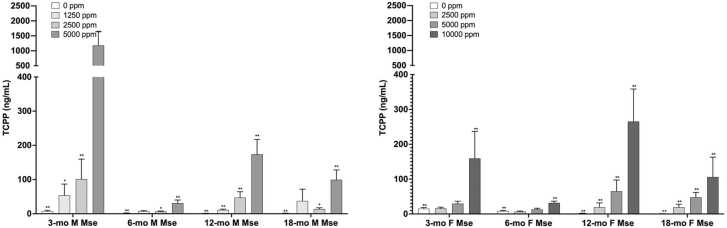
Mean TCIPP concentrations in male and female mouse plasma from 4 time points during the 2-year study. M=Male; F=Female, Mse = Mouse. Statistical analyses performed by Jonckheere (trend) and Shirley or Dunn (pairwise) tests. Statistical significance for the control group indicates a significant trend test. Statistical significance for a treatment group indicates a significant pairwise test compared to the vehicle control group. *Statistically significant at p ≤ 0.05; **Statistically significant at p ≤ 0.01. Data shown: mean ± sd; n = 4–5.

Aliquots of plasma collected from male and female mice at four TCPP exposure levels (1250 (male), 2500, 5000, and 10,000 (female) ppm) plus controls at 3-, 6-, 12-, and 18-month time points were analyzed upon receipt using the procedure described above. Mean TCIPP concentrations are shown in ([Mouse PA48]). Mean TCIPP concentrations in exposed mice ranged from 6.52 to 1180 ng/mL in male mice and 6.60 – 265 ng/mL in female mice and were significantly different from controls (p ≤ 0.01) at each time point in all exposure groups at the 12-, and 18-month time points but only in the high exposure group at 3- and 6-months. (Fig. 4). An increasing trend of TCIPP concentration with TCPP exposure concentration was observed at all time points in both male and female mice (p ≤ 0.01). At the two TCPP exposures common to male and female mice (2500 and 5000 ppm) mean TCIPP concentrations ranged from 6.52 to 1180 ng/mL and. 6.60–65.1 ng/mL in males and females, respectively. Like rats, TCIPP concentrations seen in mice at 6 and 18 months tended to be lower than those at 3 and 12 months and no consistent increase or decrease in TCIPP concentrations was observed in any TCPP-exposed group over the course of the study.

In general, mouse TCIPP plasma concentrations appeared to be more variable than those in rats. The higher variability in plasma concentrations also contributed to the lack of significant differences noted between controls and low to mid-concentration TCPP-exposed mice. It was difficult to attribute the higher variability in TCIPP concentrations to differences in food consumption in the mice because increased food spillage in the mouse study made food consumption estimates unreliable. No difference was evident in TCIPP plasma concentrations between male and female mice but was slightly higher than rats at the same TCPP exposures perhaps due to increased apparent TCPP intake in the mice.

Low concentrations of TCIPP (≤ 8.6 ng/mL) were seen in male and female control rats at the 3-, 12-, and 18-month time points and at all time points in mice. In control rat plasma, TCIPP was seen in 44 of 77 (57%) controls with individual concentrations ranging from ~10 to ~69% of those in the low exposure group ([Rat IAD48]). In mice, TCIPP was detected in 26 of 39 (67%) control samples with concentrations ranging from ≤ 10–125% of the low exposure group ([Mouse IAD48]). To assess whether the TCIPP seen in plasma from control animals resulted from exposure to TCPP in feed, we analyzed control diet from each time point for TCIPP but found no TCIPP present above the limit of quantitation (LOQ, 5 ng/mL, data not shown).

### 3.3 Plasma BCPCP concentrations

To further investigate the nature of TCIPP levels seen in control animals, we developed and qualified an LC-MS/MS method (Supplemental Table S3) to quantify three isomers of BCPCP in HSD female rat plasma. BCPCP is a Phase 1 TCPP metabolite, which in theory, should only be seen at quantifiable levels in live animals exposed to TCPP. The method was linear (r ≥ 0.99) over the range of 30–10,000 ng/mL, the precision (estimated as relative standard deviation, RSD) and accuracy (estimated as relative error, RE) were ≤ 1.8%, and ≤ ± 2.9%, respectively. The LOQ was 30 ng/mL and the LOD (1.8 ng/mL) was determined as three times the standard deviation of the baseline noise expressed as concentration. Absolute recovery ranged from 115% to 167%. Selectivity was demonstrated with BCPCP responses in matrix blanks with and without IS ≤ 7.6% of the LOQ standard response. A representative standard curve is shown in Fig. S6A. Representative chromatograms showing BCPCP response in plasma matrix standards and samples are shown in Fig. S6B.

Control plasma samples from the 6-, 12- and 18-month (rats) and 12- and 18-month (mice) time points were analyzed for BCPCP. In control rats, BCPCP concentrations at or above the LOD (1.8 ng/mL) were measured in plasma from 24 of 54 samples (44%) and in 5 of 18 samples for control mice but mean BCPCP concentrations in these samples were ≤ ~7% and ~2% of the lowest BCPCP concentration seen in TCPP-exposed rats and mice, respectively (Table 1).

**Table 1:** BCPCP Concentrations in Rat and Mouse Plasma.

Time point (Exposure Group)	Male rat	Female rat	Male mouse	Female mouse
6-month (0 ppm)	3.45 ± 5.54 [10]^a^	0.027 ± 0.081 [9]^b^	ND	ND
12-month (0 ppm)	13.7 ± 22.8 [9]	5.89 ± 7.49 [10]	328 ± 709 [5]	1.63 ± 1.39 [4]^b^
12-month (1250 ppm)	NA	NA	2152 ± 1646 [5]	ND
12-month (2500 ppm)	ND	2913 ± 1144 [9]	6276 ± 3651 [5]	ND
12-month (5000 ppm)	ND	7195 ± 2180 [10]	9683 ± 1178 [4]	ND
12-month (10,000 ppm)	ND	11,963 ± 3791 [9]	ND	ND
12-month (20,000 ppm)	ND	19,006 ± 6051 [7]	ND	ND
18-month (0 ppm)	20.4 ± 54.8 [8]	0.001 ± 0.003 [8]^b^	0.990 ± 0.326 [4]^b^	0.00 ± 0.00 [5]^b^

^a^Mean ± standard error [number]

^b^Values below analytical limit of detection (LOD) were assigned a value of ½ LOD (1.8 ng/mL) when > 20% of values in a given group were above LOD; NA: Not applicable; ND: Not determined.

To determine whether the TCIPP and BCPCP concentrations seen in the controls could have resulted from TCPP exposure, the ratio of BCPCP to TCIPP was calculated for all control samples with TCIPP and BCPCP values. The control ratios were then compared to the BCPCP:TCIPP ratios calculated for TCPP-exposed groups (Supplemental Table S4) since, if metabolism kinetics are linear, BCPCP arising from metabolism should result in a similar BCPCP:TCIPP ratio regardless of exposure level. In rats, BCPCP:TCIPP ratios could not be calculated for any of the 6- or 18-month controls because TCIPP concentrations were below the limit of detection for all but one female rat and that rat had no detectable BCPCP concentration. At the 12-month time point, TCIPP and BCPCP concentrations were measurable in 19 of 19 and 16 of 19 control samples, respectively and BCPCP:TCPP ratios ranged from 0.28 to 11.9. In contrast, BCPCP ratios for TCPP-exposed animals ranged from 82.7 to 1480. Mean ratios in rats for exposed groups were 274, 416, 386, and 492 for the 2500, 5000, 10,000, and 20,000 ppm groups compared to 2.40 for controls. In control mice (Supplemental Table S5), BCPCP:TCIPP ratios could not be calculated for any 18-month mice because no mice with detectable TCIPP or BCPCP concentrations had detectable concentrations of both analytes. In male and female control mice at the 12-month time point, detectable concentrations of TCIPP were seen in 5 of 10 mice and BCPCP could be measured in 3 of these. One male control mouse had a BCPCP concentration that resulted in a ratio of 556. Based on the low frequency of TCPP and BCPCP detection in the mouse and rat control samples and the fact that this value is similar the mean ratio of the 5000-ppm exposure group (416) (Supplemental Table S4, S5) it is likely that this mouse sample was mislabeled during sample collection or analysis. Hence, we dropped this value from consideration for further analyses. For the remaining two male mouse controls with TCIPP and BCPCP concentrations the BCPCP:TCIPP ratios were 0.813 and 43.7 in these controls compared to mean ratios of 79.6–258 for TCPP-exposed mice. The low BCPCP:TCIPP ratios seen in rats and mice, combined with the presence of TCPP without BCPCP and the presence of BCPCP without any TCPP in many samples, indicates that the TCPP concentrations seen in some controls likely arose from contamination during sample collection and/or analysis.

**Fig. 5:**
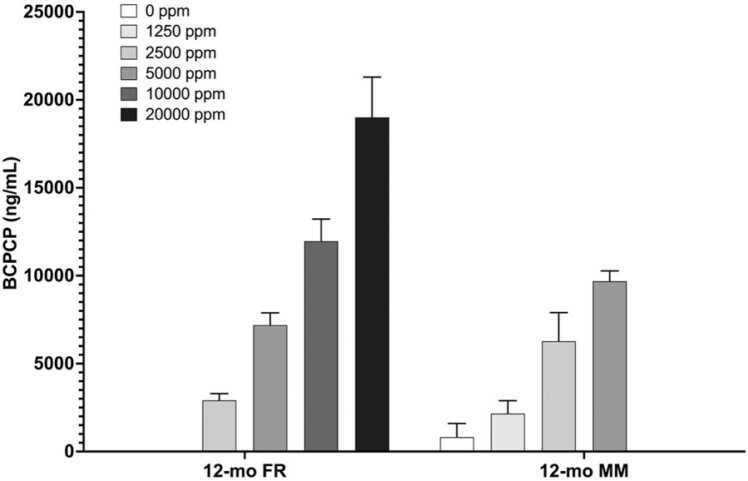
BCPCP Concentrations (ng/mL) in rat and mouse plasma at the 12-month time point. FR=Female Rat; MM=Male Mouse. Rat: n = 7–10; Mice: n = 4–5.

Samples from all female HSD rats and male B6C3F1/N mice were evaluated for BCPCP at the 12-month time point. Mean concentrations in TCPP-exposed animals (Table 1) were much higher than those in controls. In female rats, mean BCPCP plasma concentrations ranged from 2910 to 19,000 ng/mL in the 2500–20,000 ppm exposed groups compared to ~6 ng/mL in the control (Fig. 5). Female rat plasma BCPCP concentrations in all groups were approximately proportional to the TCPP exposure as evident by exposure concentration-normalized responses between 0.95 and 1.4 ng/mL/ppm. In male mice, mean BCPCP concentrations ranged from 2150 to 9680 ng/mL for the 1250–5000 ppm groups at the 12-month time point and 328 ng/mL in controls (Fig. 5). BCPCP concentrations were approximately proportional to the TCPP exposure as evident by normalized responses between 1.7 and 2.5 ng/mL/ppm. Plasma BCPCP concentrations in mice were higher than those in rats fed the same TCPP concentration demonstrating an apparent species-related effect.

## 4 Discussion

To evaluate the chronic toxicity of TCPP more comprehensively in preclinical rodent models, the DNTP incorporated determination of TCIPP in plasma to assess systemic exposure to TCPP in the study design. As such, two cohorts of animals (see methods for study design) were evaluated in parallel. Data collected from the primary cohort of rats and mice, used to evaluate the carcinogenic potential of TCPP, are not presented in this manuscript but are available at https://ntp.niehs.nih.gov/go/peerreview. A second cohort of rats and mice were specifically assigned for the measurement of the most abundant TCPP isomer (tris(1-chloro-2-propyl)phosphate, TCIPP) as representative of TCPP concentrations in plasma collected from HSD rat dams and offspring at PND28; adult male and female HSD rats at 3, 6, 12 and 18 months post-weaning; and from adult male and female B6C3F1/N mice 3, 6, 12 and 18 months after study start. Rat maternal survival, clinical observations, feed consumption, and body weights during gestation and lactation periods were evaluated in the second cohort and in male and female offspring during the lactation period. Overall, TCPP was not overtly toxic to dams or offspring exposed via feed during the perinatal period. After weaning on PND28, rats were monitored over an additional 2 years for general toxicity while plasma samples were collected and analyzed for systemic exposure assessments. Similarly, adult male and female mice were monitored for clinical signs, food consumption, and body weight changes during 2-years of exposure. TCPP did not affect the survival of adult rats and mice and clinical signs of toxicity were not observed. Decreased feed consumption and body weights were observed at higher exposure concentrations in rats and to a greater extent in mice. Despite the decreased food consumption, the estimated consumption of TCPP in both species was approximately linear with the exposure concentrations provided in feed; albeit mice appeared to consume more TCPP than rats.

On PND28, TCIPP concentrations in rats and their offspring increased with increasing exposure concentration. In male and female offspring, the TCIPP concentrations at exposure concentrations up to 10,000 ppm were proportional to TCPP exposure concentration; however, at 20,000 ppm, TCIPP concentration was more than proportional suggesting that the metabolic capacity of the offspring may have been exceeded. TCIPP levels in offspring were also higher than those in dams on PND28 possibly resulting from lower metabolic competence at this stage of development and/or co-exposure to TCPP from milk and direct consumption of feed. We also observed that TCIPP levels in dams from the PND28 time point were lower than those seen at some of the other time points, with levels at the high exposure concentration on PND28 roughly 30–40% of those at the 3- or 12-month time points. The biological significance of this result is unclear, because the half-life of TCIPP in plasma is not known, but we speculate that it may result from the increased time required to collect samples on the day of weaning relative to the half-life of TCPP in plasma. Although this study did not specifically evaluate the disposition of TCPP in vivo, other reports have shown TCPP to rapidly convert to isomers of bis(2-chloroisopropyl)phosphate, and hydroxylated and carboxylated TCPP metabolites in human liver microsomes [24], [3] suggesting a short elimination half-life for TCIPP in vivo. Studies are needed to better understand the metabolism, disposition, and underlying kinetics of TCPP and its isomers.

Post-weaning, TCIPP levels in plasma of male and female rats showed increases that were not proportional to TCPP exposure concentration at all time points. In mice TCIPP plasma levels tended to be proportional to TCPP exposure between the low and mid exposure concentrations and more than proportional at the highest exposure concentration. Over the course of the study, exposure-normalized TCIPP plasma levels in rats and mice remained approximately the same within each exposure group suggesting that bioaccumulation is not occurring with repeat dietary exposure. Exposure-normalized TCIPP concentrations showed no differences between male and female rats and female mice, but male mice had higher TCIPP concentrations, suggesting a difference in TCIPP absorption and/or clearance between male and female mice.

TCIPP was detected in some control rats and mice. To assess the source of the TCIPP we determined the concentration of BCPCP, a Phase 1 TCPP metabolite, which should theoretically be present in measurable quantities only in animals exposed to TCPP in vivo. Measured BCPCP concentrations were compared to TCIPP concentrations in the same animal by calculating BCPCP:TCIPP ratios. BCPCP:TCIPP ratios in control rats and mice compared to those in exposed animals suggest that detectable TCIPP concentrations in controls resulted from contamination during sampling and/or analysis due to the presence of background TCPP levels in the built environment [10], [20], [28], [29], [30], [31], [32] has shown that other ubiquitous environmental chemicals, including bisphenol A, triclosan, and parabens, can contaminate reagents and analytical standards during analysis. Thus, when TCIPP levels were detected in samples that showed no detectable BCPCP it is likely due to contamination by TCPP present in the testing or analytical laboratories during sample collection or preparation.

BCPCP levels measured in all exposure groups of female rats and male mice at the 12-month time point were 1–3 orders of magnitude higher than the TCIPP concentrations at each exposure concentration and were approximately proportional to TCPP exposure concentrations. BCPCP levels in rats were higher than those in mice in all exposure groups which is consistent with the relatively higher TCIPP concentrations seen in mice, suggesting less metabolism of TCIPP to BCPCP in mice. Sex differences could not be evaluated because only one sex was assessed in each species. To our knowledge, this is one of the first exposure studies characterizing the BCPCP metabolite following continual TCPP exposure. In our study, the significantly higher levels of BCPCP compared to TCPP show that this metabolite could be added to the list of acceptable biomarkers of TCPP exposure (i.e., BCPP and BCIPHIPP) [23], [24]; ; ([33]Gibson et al., 2019); [8]. We suggest that future studies of TCPP systematically evaluate BCPCP alongside other major metabolites following chemical exposure to enable better comparisons across datasets.

## 5 Conclusion

TCIPP, the primary isomer of TCPP, was quantified in rats and mice exposed to TCPP for 2 years through feed. In rats and mice of both sexes, TCIPP concentrations were less than proportional to the exposure concentration within a time point, possibly indicating changes in ADME processes with increasing exposure concentration. TCIPP concentrations in mice were slightly higher than those in rats at all time points suggesting a potential species difference. There were no sex differences in rats. In mice, a small difference in TCIPP concentrations between males and females was noted, with male mice having consistently higher plasma concentrations than females. TCIPP concentrations did not appear to increase over the course of the study suggesting that no bioaccumulation was occurring. Interestingly, low concentrations of TCIPP were seen in 57% of rat and 67% of mouse controls. Following a comparison of TCIPP and BCPCP concentrations in control and selected TCPP-exposed animals, we hypothesize that low levels of TCIPP seen in controls likely resulted from the presence of low background TCPP levels during sample collection, preparation, and/or analysis.

## CRediT authorship contribution statement

**Bradley J. Collins**: Methodology, Project administration, Supervision, Data curation, Formal analysis, Visualization, Writing – original draft preparation, Writing – review and editing. **Desmond Slade**: Methodology, Investigation, Data curation, Visualization, Writing – review and editing. **Kristin Aillon**: Supervision, Writing – review and editing. **Matt Stout**: Project conceptualization, Project administration, Writing – review and editing. **Laura Betz**: Data curation, Formal analysis-statistical analysis. **Suramya Waidyanatha**: Conceptualization, Methodology, Supervision, Writing – review and editing. **Kristen Ryan**: Project administration, Data curation, Formal analysis, Writing – original draft preparation, Writing – review and editing.

## Declaration of Competing Interest

The authors declare that they have no known competing financial interests or personal relationships that could have appeared to influence the work reported in this paper.
