# Supplementary material for: Plasma concentrations of tris(1-chloro-2-propyl) phosphate and a metabolite bis(2-chloroisopropyl) 1-carboxyethyl phosphate in Sprague-Dawley rats and B6C3F1/N mice from a chronic study of tris(chloropropyl) phosphate via feed
Source: Toxicol Rep. 2022 Mar 29;9:690–8. doi: 10.1016/j.toxrep.2022.03.025 (PMC9010517; doi:10.1016/j.toxrep.2022.03.025)
Supplement: Supplementary file 1 — Supplementary material [file mmc1.docx]

BCPCP Synthesis Method

BCPCP was prepared by reacting one equivalent (±)-ethyl lactate (400 mmole) with one equivalent phosphorus oxychloride (400 mmole) at –20°C in the presence of triethylamine (TEA) (400 mmole) in 100 mL of tetrahydrofuran (THF), followed by overnight stirring (–20°C → room temperature (r.t.), yielding 1-carbethoxyethyl phosphorodichloridite (Lot No. 12861-5-18).

The resulting dichloro ethyl ester intermediate was filtered and reacted with two equivalents 1-chloro-2-propanol6 (800 mmole) at –20°C in the presence of 4-dimethylaminopyridine (DMAP) (800 mmole) in methylene chloride (CH_2_Cl_2_), followed by stirring for two days (–20°C → r.t.). The resulting dichloropropyl ethyl ester intermediate was filtered, and the organic solvent removed, yielding bis(2-chloroisopropyl) 1-carbethoxyethyl phosphate (Lot No. 12861-12-20), a light orange crude oil.

A portion of the crude product (6.0 g, 17.1 mmole) was dissolved in 60 mL of methanol (MeOH), cooled to 5°C, and hydrolyzed by dropwise addition of aqueous sodium carbonate (Na_2_CO_3_) (283 mM, 60 mL) at 5°C, followed by overnight stirring (5°C [6 hours] → r.t.). Workup of the reaction included adding 200 mL of water to the mixture, followed by salt (NaCl) to saturation, and extraction with ethyl acetate (EtOAc). The resulting aqueous layer was acidified to pH ~ 2 and extracted with EtOAc. The combined organic fraction was dried over magnesium sulfate (MgSO_4_) and the organic solvent removed to yield the final product, a straw-colored viscous oil. The final hydrolysis step was repeated an additional three times using Lot No. 12861-12-20 to provide ~11.0 g of the final product, bis(2-chloroisopropyl) 1-carboxyethyl phosphate (BCPCP, Lot No. 12861-16-23).

Supplemental Tables

Table S1. GC-FID Instrument System and Parameters for BCPCP Purity Determination

| Instrument: | Agilent 6890 gas chromatograph |
| --- | --- |
| Column: | J&W Scientific DB-1, 30 m × 0.32 mm ID, 0.25 µm film thickness |
| Injector Temperature: | 200°C |
| Injector Mode: | Splitless |
| Detector: | Flame Ionization Detector (FID) |
| Detector Temperature: | 250°C |
| Carrier Gas/Flow Rate: | Helium: ~ 1.1 mL/min |
| Injection Volume: | 1, 2 µL |
| Oven Program | 60°C (hold 0 min), 10°C/min ramp to 240°C (hold 7 min) |

Table S2. GC-FID Instrument System and Parameters for the analysis of TCPP in NTP-2000 Feed

| Instrument: | Agilent 6890 gas chromatograph |
| --- | --- |
| Column: | J&W Scientific DB-5, 30 m × 0.53 mm ID, 1.5 µm film thickness |
| Injector Temperature: | 250°C; purge on at 0.75 min |
| Injector Mode: | Splitless |
| Detector: | Flame Ionization Detector (FID) |
| Detector Temperature: | 300°C |
| Carrier Gas/Flow Rate: | Helium: ~10.0 mL/min |
| Hydrogen Flow: | ~30 mL/min |
| Air Flow: | 300 mL/min |
| Make-up Gas: | Nitrogen: ~20 mL/min |
| Injection Volume: | 1.0 µL |
| Oven Program | 160°C (hold 5 min), 1.0°C/min ramp to 180°C (hold 5 min) |

Table S3. LC-MS/MS Instrument System and Parameters for BCPCP Quantitation in Plasma

| Liquid Chromatograph (LC) | Shimadzu Scientific Instruments HPLC with SCL-10Avp system controller and LC-10ADvp solvent delivery unit |
| --- | --- |
| Autosampler | Leap Technologies (CTC Analytics) HTC PAL autosampler |
| Temperature  Injection Volume | 5°C  10 µL |
| Switching Valve | Shimadzu FCV-12AH |
| Column | Phenomenex Kinetex 2.6 µm PFP 100 Å, 50 × 2.1 mm |
| Guard Column  Column Temperature  Flow Rate | Phenomenex SecurityGuard ULTRA PFP, 2 × 2.1 mm  35°C  0.25 mL/min |
| Mobile Phase A  Mobile Phase B | DI H_2_O with 0.1% formic acid  MeOH with 0.1% formic acid |
| LC Gradient Program | 80% A to 5% A (4 min, hold 3 min), 5% A to 80% A (1 min, hold 2 min) |
| Switching Valve Program | 0 to 2 min (flow to waste), 2 to 8.25 min (flow to source), 8.25 to 10 min (flow to waste) |
| Mass Spectrometer (MS) | Applied Biosystems SCIEX API-4000 Q-TRAP MS |
| Ionization Source | Positive (ESI) TurboIonSpray (TIS) |
| Curtain Gas (CUR) | 10 psi (Nitrogen) |
| Collision Gas Setting (CAD) | 4 |
| Ionization Voltage (IS) | 5,500 C |
| Source Temperature (TEM) | 400ºC |
| Ion Source Gas GS1/GS2 | 30 psi / 35 psi |
| Interface Heater (IHE) | On |
| Entrance Potential (EP) | 10 V |
| Declustering Potential (DP) | 71 V (BCPCP) / 66 V (DBzP) |
| Collision Energy (CE) | 21 V (BCPCP) / 25 V (DBzP) |
| Collision Cell Exit Potential (CXP) | 14 V (BCPCP) / 6 V (DBzP) |
| Scan Mode | Multiple reaction monitoring (MRM) |
| MRM (*m/z*) | BCPCP: 323.063 → 170.884 Da (quantitation)  BCPCP: 323.063 → 99.024 Da (confirmation)  DBzP: 279.129 → 91.111 Da |
| Retention Times | BCPCP: ~3.75 min (isomers co-elute as a single peak)  DBzP (IS): ~4.35 min (IS) |
| Data Analysis | SCIEX Analyst software, version 1.5.1 |

PFP: Pentafluorophenyl with TMS endcapping.

Table S4. BCPCP/TCIPP Ratios in Rat Plasma

|  | 6-Month | | | | | 12-month | | | | | 18-month | | | | |
| --- | --- | --- | --- | --- | --- | --- | --- | --- | --- | --- | --- | --- | --- | --- | --- |
| Species | Animal No. | TCPP Exposure (mg/kg) | TCIPP (ng/mL) | BCPCP^1^ (ng/mL) | BCPCP/TCIPP Ratio | Animal No. | TCPP Exposure (mg/kg) | TCIPP (ng/mL) | BCPCP^1^ (ng/mL) | BCPCP/TCIPP Ratio | Animal No. | TCPP Exposure (mg/kg) | TCIPP (ng/mL) | BCPCP^1^ (ng/mL) | BCPCP/TCIPP Ratio |
| Male Rat^2^ | 601 | 0 | BLOD^3^ | BLOD | N/A^4^ | 601 | 0 | 3.837 | 10.65 | 2.78 | 601 | 0 | BLOD | BLOD | N/A |
|  | 602 | 0 | BLOD | 17.73 | N/A | 602 | 0 | 2.797 | 13.75 | 4.92 | 602 | 0 | BLOD | BLOD | N/A |
|  | 603 | 0 | BLOD | 2.526 | N/A | 603 | 0 | 2.876 | 0.800 | 0.28 | 603 | 0 | 1.529 | 0.157 | 0.103 |
|  | 604 | 0 | BLOD | BLOD | N/A | 604 | 0 | 5.017 | 10.06 | 2.00 | 604 | 0 | BLOD | BLOD | N/A |
|  | 605 | 0 | BLOD | BLOD | N/A | 605 | 0 | 4.051 | 11.928 | 2.94 | 605 | 0 | 1.808 | BLOD | N/A |
|  | 606 | 0 | BLOD | BLOD | N/A | 606 | 0 | --^5^ | -- | N/A | 606 | 0 | -- | -- | N/A |
|  | 607 | 0 | BLOD | 8.837 | N/A | 607 | 0 | 3.347 | 1.587 | 0.47 | 607 | 0 | 1.397 | 155.87 | 111.57 |
|  | 608 | 0 | BLOD | BLOD | N/A | 608 | 0 | 2.926 | BLOD | N/A | 608 | 0 | BLOD | BLOD | N/A |
|  | 609 | 0 | BLOD | 0.522 | N/A | 609 | 0 | 2.932 | BLOD | N/A | 609 | 0 | BLOD | BLOD | N/A |
|  | 610 | 0 | BLOD | BLOD | N/A | 610 | 0 | 6.125 | 72.931 | 11.91 | 610 | 0 |  |  |  |
| Female Rat^6^ | 651 | 0 | BLOD | BLOD | N/A | 651 | 0 | 2.445 | 24.85 | 10.16 | 651 | 0 | BLOD | BLOD | N/A |
|  | 652 | 0 | BLOD | 0.242 | N/A | 652 | 0 | 2.799 | 4.400 | 1.57 | 652 | 0 | -- | -- | N/A |
|  | 653 | 0 | BLOD | BLOD | N/A | 653 | 0 | 6.015 | 6.524 | 1.08 | 653 | 0 | BLOD | 0.00781 | N/A |
|  | 654 | 0 | BLOD | BLOD | N/A | 654 | 0 | 4.131 | 5.241 | 1.27 | 654 | 0 | 0.996 | BLOD | N/A |
|  | 655 | 0 | BLOD | --^4^ | N/A | 655 | 0 | 2.817 | 1.113 | 0.40 | 655 | 0 | 2.855 | BLOD | N/A |
|  | 656 | 0 | 1.405 | BLOD | N/A | 656 | 0 | 1.33 | 0.721 | 0.54 | 656 | 0 | 1.846 | BLOD | N/A |
|  | 657 | 0 | BLOD | BLOD | N/A | 657 | 0 | 1.376 | BLOD | N/A | 657 | 0 | 3.591 | BLOD | N/A |
|  | 658 | 0 | BLOD | BLOD | N/A | 658 | 0 | 2.352 | 11.40 | 4.85 | 658 | 0 | BLOD | BLOD | N/A |
|  | 659 | 0 | BLOD | BLOD | N/A | 659 | 0 | 2.379 | 0.933 | 0.39 | 659 | 0 | -- | -- | N/A |
|  | 660 | 0 | BLOD | BLOD | N/A | 660 | 0 | 2.173 | 2.857 | 1.31 | 660 | 0 | BLOD | BLOD | N/A |
|  |  |  |  |  |  | 661 | 2500 | 32.66 | 5678.7 | 173.9 |  |  |  |  |  |
|  |  |  |  |  |  | 662 | 2500 | 9.867 | 3257.1 | 330.1 |  |  |  |  |  |
|  |  |  |  |  |  | 663 | 2500 | 46.04 | 3809.8 | 82.7 |  |  |  |  |  |
|  |  |  |  |  |  | 664 | 2500 | 4.589 | 2781.9 | 606.2 |  |  |  |  |  |
|  |  |  |  |  |  | 665 | 2500 | -- | -- | N/A |  |  |  |  |  |
|  |  |  |  |  |  | 666 | 2500 | 10.29 | 2237.6 | 217.5 |  |  |  |  |  |
|  |  |  |  |  |  | 667 | 2500 | 17.9 | 2865.4 | 160.1 |  |  |  |  |  |
|  |  |  |  |  |  | 668 | 2500 | 5.523 | 2631.1 | 476.4 |  |  |  |  |  |
|  |  |  |  |  |  | 669 | 2500 | 23.39 | 2406.7 | 102.9 |  |  |  |  |  |
|  |  |  |  |  |  | 670 | 2500 | 5.213 | 1544.5 | 296.3 |  |  |  |  |  |
|  |  |  |  |  |  | 671 | 5000 | 82.27 | 10489 | 127.5 |  |  |  |  |  |
|  |  |  |  |  |  | 672 | 5000 | 29.21 | 7572 | 259.2 |  |  |  |  |  |
|  |  |  |  |  |  | 673 | 5000 | 43.31 | 6447.4 | 148.9 |  |  |  |  |  |
|  |  |  |  |  |  | 674 | 5000 | 21.08 | 4028.3 | 191.1 |  |  |  |  |  |
|  |  |  |  |  |  | 675 | 5000 | 12.29 | 4678.5 | 380.7 |  |  |  |  |  |
|  |  |  |  |  |  | 676 | 5000 | 12.1 | 5769.5 | 476.8 |  |  |  |  |  |
|  |  |  |  |  |  | 677 | 5000 | 33.44 | 10082 | 301.5 |  |  |  |  |  |
|  |  |  |  |  |  | 678 | 5000 | 4.104 | 6077 | 1480.8 |  |  |  |  |  |
|  |  |  |  |  |  | 679 | 5000 | 16.77 | 8807.8 | 525.2 |  |  |  |  |  |
|  |  |  |  |  |  | 680 | 5000 | 29.5 | 7992.9 | 270.9 |  |  |  |  |  |
|  |  |  |  |  |  | 681 | 10000 | 8.088 | 3388.6 | 419.0 |  |  |  |  |  |
|  |  |  |  |  |  | 682 | 10000 | 11.04 | 3520.2 | 318.9 |  |  |  |  |  |
|  |  |  |  |  |  | 683 | 10000 | -- | -- | N/A |  |  |  |  |  |
|  |  |  |  |  |  | 684 | 10000 | 9.781 | 6297.9 | 643.9 |  |  |  |  |  |
|  |  |  |  |  |  | 685 | 10000 | 8.765 | 9054.6 | 1033.0 |  |  |  |  |  |
|  |  |  |  |  |  | 686 | 10000 | 40.53 | 6723.7 | 165.9 |  |  |  |  |  |
|  |  |  |  |  |  | 687 | 10000 | 21.35 | 3815.7 | 178.7 |  |  |  |  |  |
|  |  |  |  |  |  | 688 | 10000 | 24.51 | 4144.2 | 169.1 |  |  |  |  |  |
|  |  |  |  |  |  | 689 | 10000 | 27.6 | 4431.4 | 160.6 |  |  |  |  |  |
|  |  |  |  |  |  | 690 | 10000 | 29.04 | 11079 | 381.5 |  |  |  |  |  |
|  |  |  |  |  |  | 691 | 20000 | 21.19 | 5112.1 | 241.3 |  |  |  |  |  |
|  |  |  |  |  |  | 692 | 20000 | 10.92 | 5782.4 | 529.5 |  |  |  |  |  |
|  |  |  |  |  |  | 693 | 20000 | 7.044 | 9766.0 | 1386.4 |  |  |  |  |  |
|  |  |  |  |  |  | 694 | 20000 | 337.3 | 9755.5 | 28.90 |  |  |  |  |  |
|  |  |  |  |  |  | 695 | 20000 | 16.18 | 7209.7 | 445.6 |  |  |  |  |  |
|  |  |  |  |  |  | 696 | 20000 | 14.37 | 5468.2 | 380.5 |  |  |  |  |  |
|  |  |  |  |  |  | 697 | 20000 | 17.86 | 7676.6 | 429.8 |  |  |  |  |  |
|  |  |  |  |  |  | 698 | 20000 | 110.7 | -- | N/A |  |  |  |  |  |
|  |  |  |  |  |  | 699 | 20000 | 202.3 | -- | N/A |  |  |  |  |  |
|  |  |  |  |  |  | 700 | 20000 | 23.32 | -- | N/A |  |  |  |  |  |

^1^BCPCP values are reported as the sum of the response for the three measurable BCPCP isomers.

^2^Samples were not analyzed for TCPP-exposed male rats at 6, 12 or 18 months

^3^BLOD: Below the limit of detection (0.94 ng/mL TCPP; 1.8 ng/mL BCPCP)

^4^N/A: Not applicable. Ratio could not be calculated

^5^No sample received

^6^Samples were not analyzed for TCPP-exposed female rats at 6 or 18 months

Table S5. BCPCP/TCPP Ratios in Mouse Plasma

|  | 12-month | | | | | 18-month | | | | |
| --- | --- | --- | --- | --- | --- | --- | --- | --- | --- | --- |
| Species | Animal No. | TCPP Exposure (mg/kg) | TCIPP (ng/mL) | BCPCP^1^ (ng/mL) | BCPCP/TCIPP Ratio | Animal No. | TCPP Exposure (mg/kg) | TCIPP (ng/mL) | BCPCP^1^ (ng/mL) | BCPCP/TCIPP Ratio |
| Female Mouse^2^ | 589 | 0 | 1.216 | 0.989 | 0.813 | 583 | 0 | BLOD | BLOD | N/A |
|  | 595 | 0 | 1.238 | BLOD^3^ | N/A^4^ | 585 | 0 | BLOD | BLOD | N/A |
|  | 596 | 0 | BLOD | BLOD | N/A | 586 | 0 | BLOD | BLOD | N/A |
|  | 599 | 0 | 4.127 | --^5^ | N/A | 588 | 0 | 1.183 | BLOD | N/A |
|  | 600 | 0 | BLOD | 3.715 | N/A | 591 | 0 | 1.162 | BLOD | N/A |
| Male Mouse | 502 | 0 | BLOD | BLOD | N/A | 505 | 0 | 1.346 | BLOD | N/A |
|  | 506 | 0 | BLOD | BLOD | N/A | 507 | 0 | BLOD | 1.458 | N/A |
|  | 508 | 0 | 0.9724 | 42.471 | 43.68 | 510 | 0 | -- | -- | N/A |
|  | 511 | 0 | BLOD | BLOD | N/A | 512 | 0 | 2.527 | BLOD | N/A |
|  | 518 | 0 | 2.868 | 1595.9 | 556 | 516 | 0 | BLOD | BLOD | N/A |
|  | 526 | 1250 | 14.84 | 4511.5 | 304.01 |  |  |  |  |  |
|  | 531 | 1250 | 16.53 | 1652.6 | 99.98 |  |  |  |  |  |
|  | 533 | 1250 | 3.174 | 15.926 | 5.02 |  |  |  |  |  |
|  | 534 | 1250 | 6.847 | 2751.0 | 401.78 |  |  |  |  |  |
|  | 537 | 1250 | 13.58 | 1831.4 | 134.86 |  |  |  |  |  |
|  | 543 | 2500 | 34.51 | 4765.8 | 138.10 |  |  |  |  |  |
|  | 544 | 2500 | 29.05 | 3455.5 | 118.95 |  |  |  |  |  |
|  | 550 | 2500 | 4.237 | 3364.6 | 794.10 |  |  |  |  |  |
|  | 551 | 2500 | 64.32 | 7918.8 | 123.12 |  |  |  |  |  |
|  | 552 | 2500 | 103.8 | 11873 | 114.38 |  |  |  |  |  |
|  | 562 | 5000 | 329.6 | -- | -- |  |  |  |  |  |
|  | 566 | 5000 | 125.1 | 8634.1 | 69.02 |  |  |  |  |  |
|  | 569 | 5000 | 88.52 | 9622.5 | 108.7 |  |  |  |  |  |
|  | 574 | 5000 | 208.1 | 9132.4 | 43.88 |  |  |  |  |  |
|  | 578 | 5000 | 117.1 | 11342 | 96.86 |  |  |  |  |  |

^1^BCPCP values are reported as the sum of the response for the three measurable BCPCP isomers.

^2^No samples analyzed for TCPP-exposed female mice at the 18-month time point

^3^BLOD: Below the limit of detection (0.94 ng/mL TCPP; 1.8 ng/mL BCPCP)

^4^N/A: Not applicable. No ratio could be calculated.

^5^No sample received

Supplemental Figures


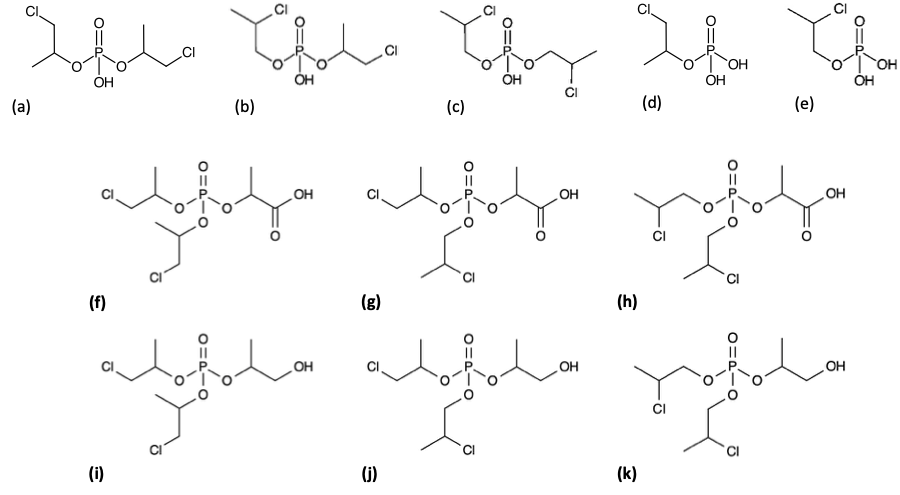


Figure S1. Structures of TCPP Metabolite Isomers.

*Bis(2-chloroisopropyl) phosphate Isomers:* (a) bis(1-chloro-2-propyl) phosphate; (b) (1-chloro-2-propyl) (2-chloro-1-propyl) phosphate; (c) bis(2-chloro-1-propyl) phosphate. *Mono(2-chloroisopropyl) phosphate Isomers:* (d) 1-chloro-2-propyl phosphate; (e) 2-chloro-1-propyl phosphate. *Bis(2-chloroisopropyl) 1-carboxyethyl phosphate Isomers:* (f) bis(1-chloro-2-propyl) 1-carboxyethyl phosphate (BCPCP-1); (g) (1-chloro-2-propyl) (2-chloro-1-propyl) 1-carboxyethyl phosphate (BCPCP-2); (h) bis(2-chloro-1-propyl) 1-carboxyethyl phosphate (BCPCP-3). *Bis(2-chloroisopropyl) 1-hydroxy-2-propyl phosphate Isomers:* (i) bis(1-chloro-2-propyl) 1-hydroxy-2-propyl phosphate; [ (j) (1-chloro-2-propyl) (2-chloro-1-propyl) 1-hydroxy-2-propyl phosphate; (k) (bis(2-chloro-1-propyl) 1-hydroxy-2-propyl phosphate].


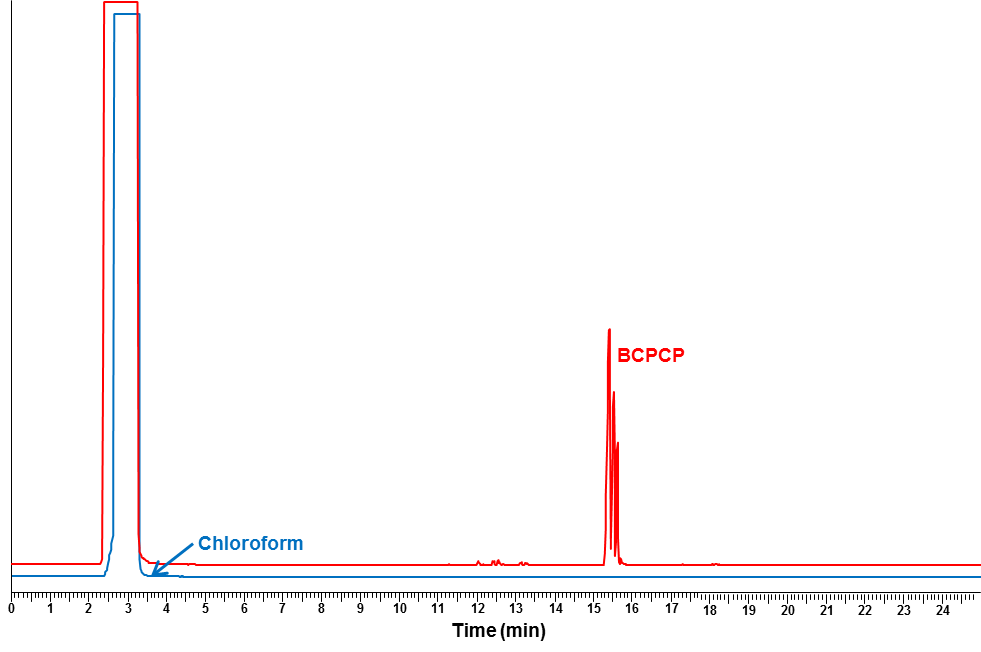


Figure S2. Representative GC-FID chromatogram for BCPCP Purity Determination. Red trace: BCPCP; Blue trace: Chloroform solvent blank.


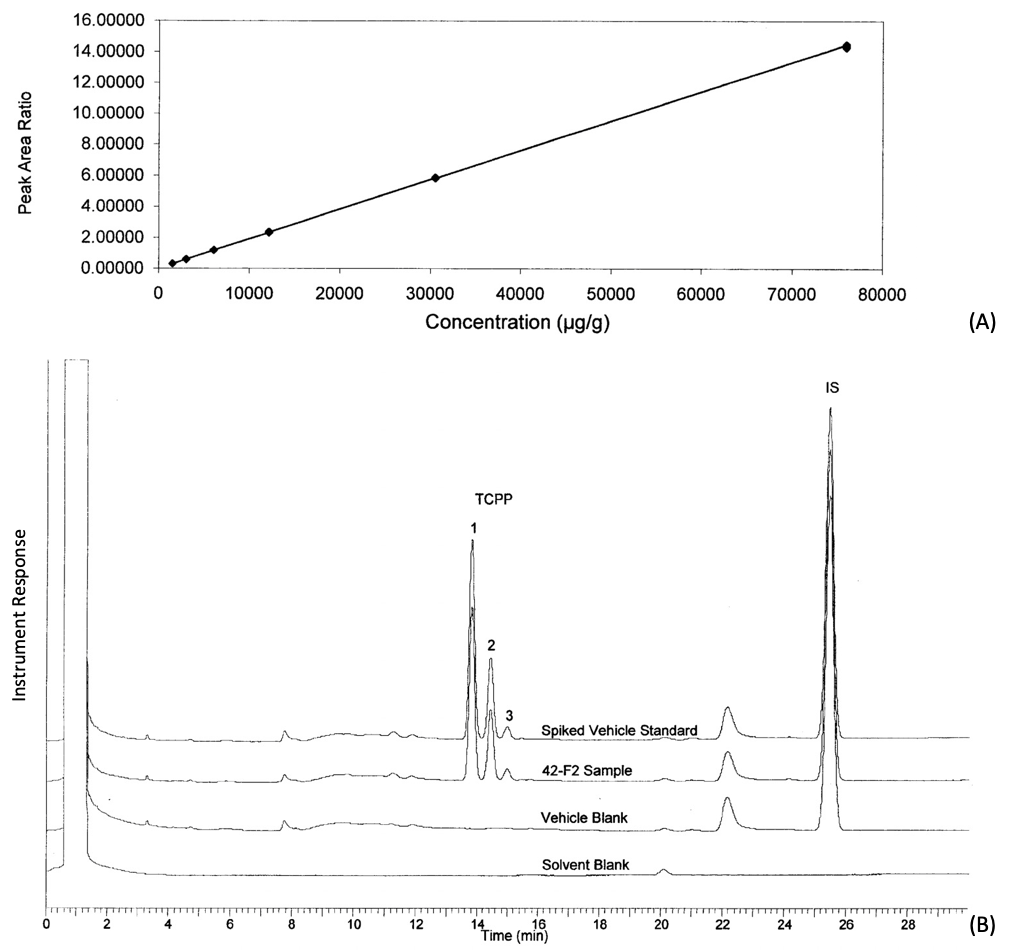


Figure S3. (A) Representative standard curve for the analysis of TCPP in NTP-2000 feed. (B) Representative GC-FID chromatograms for TCPP Determination in Feed. Showing: Solvent blank; Vehicle blank; Feed sample (42-F2); Spiked vehicle standard.

IS: Internal Standard, Isodrine (CASRN: 465-73-6); TCPP consisting of three peaks labeled 1-3.


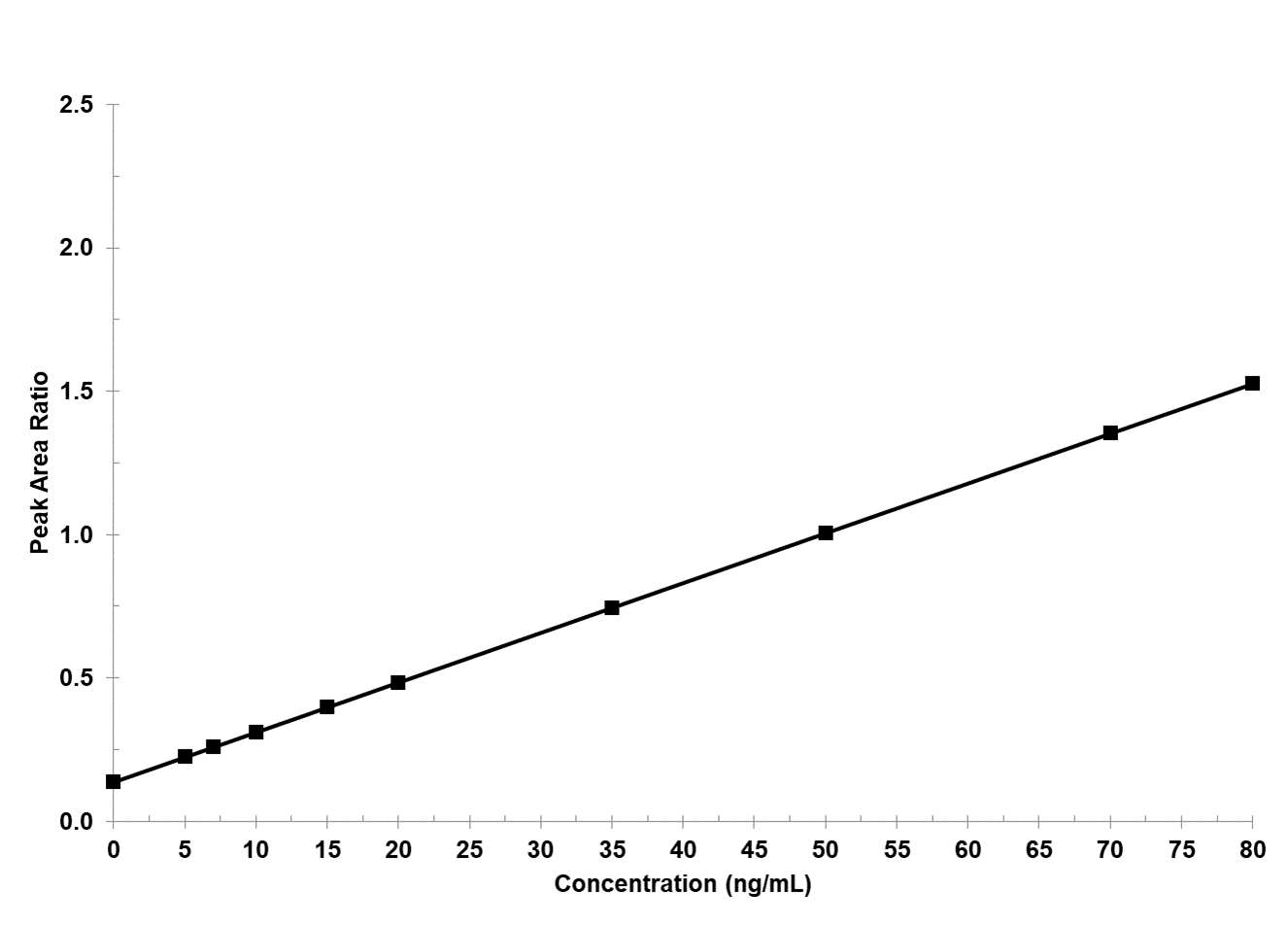


Figure S4. Representative matrix standard curve for TCIPP in plasma.


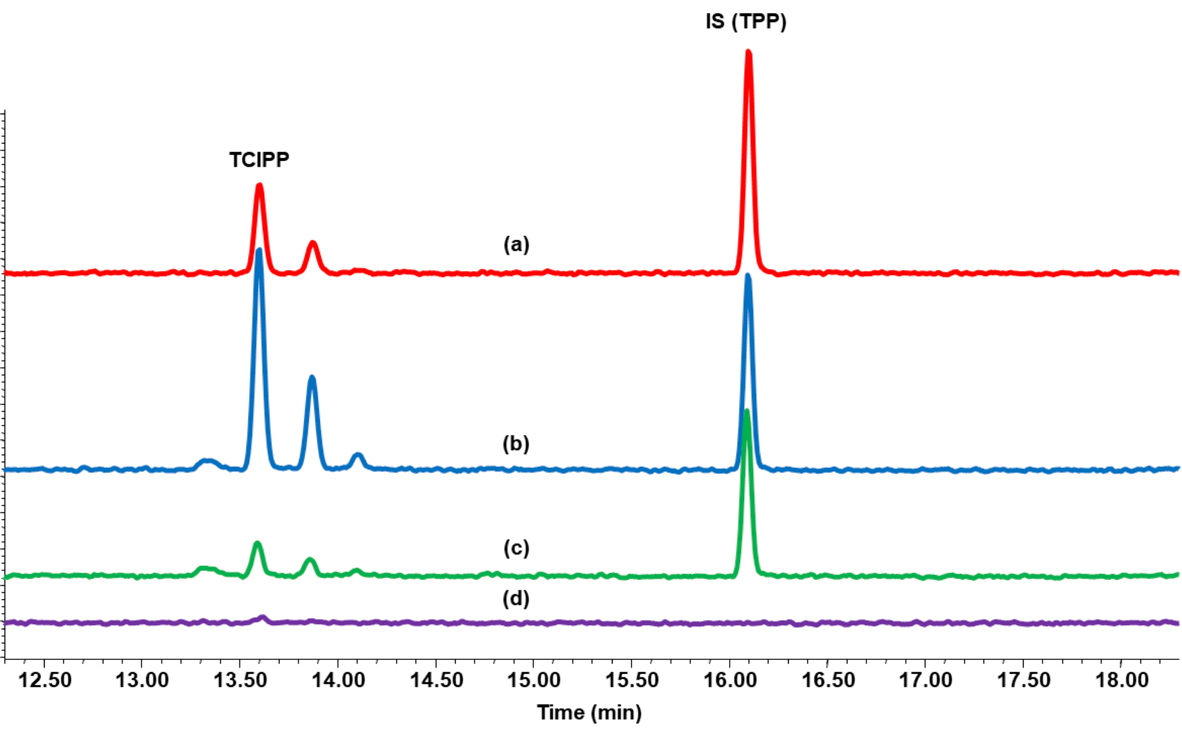


Figure S5 Representative GC-FPD chromatograms for TCIPP in plasma. (a) Matrix Standard (~ 20 ng/mL); (b) Sample from Animal 677; (c) Sample from Animal 672; (d) Matrix blank without Internal Standard (IS, Tripentyl phosphate).


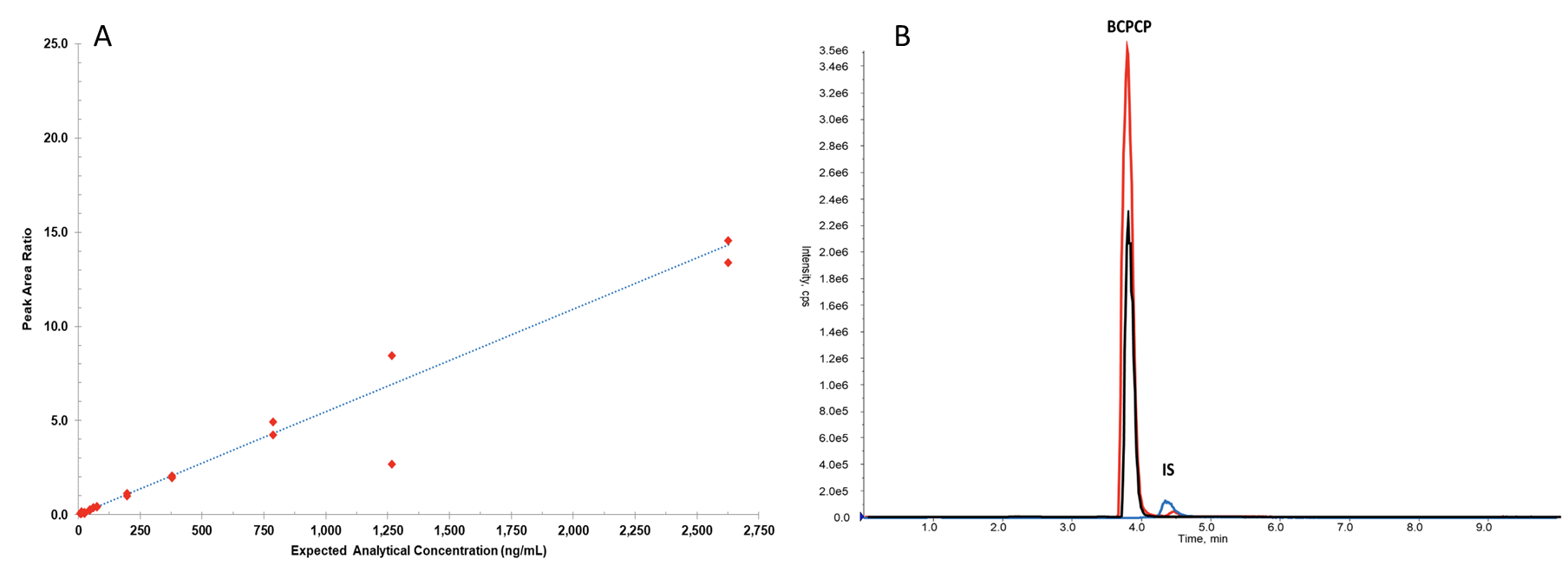


Figure S6. (A) Representative matrix standard curve for BCPCP in plasma; (B) Representative LC-MS/MS chromatograms for BCPCP in plasma (red) Matrix standard (10,000 ng/mL in plasma; 2600 ng/mL analytical); Black – Mouse Study Sample 569 (5,000 ppm); Blue – Internal Standard (IS; dibenzyl phosphate).
